# On the force field optimisation of $\beta$-lactam cores using the force field Toolkit

**DOI:** 10.1007/s10822-022-00464-3

**Published:** 2022-07-11

**Authors:** Qiyang Wu, Tianyang Huang, Songyan Xia, Frank Otto, Tzong-Yi Lee, Hsien-Da Huang, Ying-Chih Chiang

**Affiliations:** 1 Kobilka Institute of Innovative Drug Discovery, School of Medicine, The Chinese University of Hong Kong (Shenzhen), Shenzhen, 518172 China; 2 Warshel Institute for Computational Biology, School of Medicine, The Chinese University of Hong Kong (Shenzhen), Shenzhen, 518172 China; 3 Department of Chemistry, University College London, London, WC1H 0AJ UK

**Keywords:** Ligand force field parametrisation, ffTK, Dihedral phase shifts prediction, CGenFF

## Abstract

When employing molecular dynamics (MD) simulations for computer-aided drug design, the quality of the used force fields is highly important. Here we present reparametrisations of the force fields for the core molecules from 9 different $\beta$-lactam classes, for which we utilized the force field Toolkit and Gaussian calculations. We focus on the parametrisation of the dihedral angles, with the goal of reproducing the optimised quantum geometry in MD simulations. Parameters taken from CGenFF turn out to be a good initial guess for the multiplicity of each dihedral angle, but the key to a successful parametrisation is found to lie in the phase shifts. Based on the optimised quantum geometry, we come up with a strategy for predicting the phase shifts prior to the dihedral potential fitting. This allows us to successfully parameterise 8 out of the 11 molecules studied here, while the remaining 3 molecules can also be parameterised with small adjustments. Our work highlights the importance of predicting the dihedral phase shifts in the ligand parametrisation protocol, and provides a simple yet valuable strategy for improving the process of parameterising force fields of drug-like molecules.

**Supplementary Information:**

The online version contains supplementary material available at 10.1007/s10822-022-00464-3.

## Introduction

Recent years have witnessed the rise of antibiotic resistance which makes it increasingly difficult to treat bacterial infections [1–3]. In order to develop new antibiotics, it is relevant to understand how the current ones lose their effectiveness. Consequently, research related to the resistance against $\beta$-lactam antibiotics has attracted much attention in the past 20 years, including resolving structures of penicillin-binding proteins in complex with $\beta$-lactams [4], investigating the reason for bacterial death in the presence of $\beta$-lactams and vancomycin [5], as well as computational modelling of the penicillin-binding proteins [6]. A recent report also shows that 13 out of 80 antibiotics currently under development are $\beta$-lactams [7]. On the other hand, computer-aided drug design [8, 9] now is regularly used in different aspects of drug-related research. In particular, molecular dynamics (MD) simulations can help to reveal the protein-ligand interaction at an atomic level [10, 11], or to reveal the ligand binding pathway [12].

**Fig. 1:**
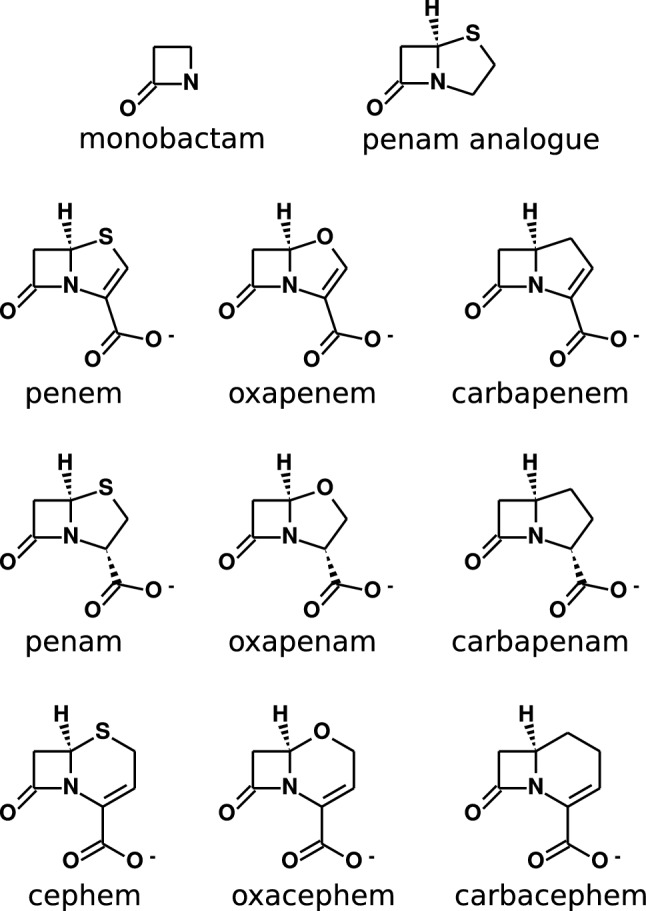
The $\beta$-lactam cores being parametrised in this work

Given the relevance of antibiotic resistance and the advances in computer-aided drug design, it is natural to employ MD simulations to investigate the interaction between penicillin-binding proteins and $\beta$-lactams [13]. Such simulations rely on good quality force fields which model the potential energy function of the whole system. However, we discovered that the often used ligand force fields, such as the CHARMM general force field (CGenFF) [14], return parameters with high penalties for antibiotics such as penicillin G and ceftaroline. Since the penalty indicates whether the parameter extracted from the force field database is a good analogy [15, 16], parameters with high penalties need to be optimised before they can be used in simulations. Thus, an intensive parameter optimisation for $\beta$-lactams is mandatory. Since there are many different $\beta$-lactam antibiotics, we first focus on the most crucial but challenging part, namely, the fused rings that characterize a $\beta$-lactam class. The structures are depicted in Fig. 1.

Many software packages have been developed to simplify the entire workflow of ligand parametrisation, e.g. force field Toolkit (ffTK) [17] and ParaMol [18]. ffTK [17] is a VMD [19] plugin designed to assist users in optimising ligand force field parameters with a friendly graphical user interface. It can generate parameters compatible with the CHARMM and CGenFF force fields [14, 20], through four major steps: the geometry optimisation, the partial atomic charge optimisation, the bond and angle optimisation, and finally the dihedral and improper dihedral angle optimisation. In each step, quantum calculations are performed and the molecular mechanics (MM) parameters are determined by fitting to the quantum data. While such software has hugely simplified the workflow, achieving a good parametrisation can still be very challenging, owing to a less well-defined dihedral parametrisation protocol. In all-atom force fields like CHARMM, dihedral angle potentials are modeled by functions of cosines [14],

1 $$\begin{aligned} V_\text {dihedrals} = \sum _{\text {dihedrals}} k_{\phi } \left[ 1 + \cos \left( n \phi - \delta \right) \right] \quad , \end{aligned}$$

where $k_{\phi }$ is the force constant for a dihedral angle $\phi$, *n* is the multiplicity ($n = 1$, 2, 3, 4, or 6), and $\delta$ is the phase shift ($\delta =0^\circ$ or $180^\circ$). Because several cosine functions with different multiplicities *n* can be combined to describe the potential of a single dihedral angle, determining the right combination based on fitting MM parameters to reproduce QM dihedral potentials can be nontrivial. Pavlova and Gumbart reported this issue when optimising the parameters of macrolides [21], antibiotics that target the protein synthesis in bacteria. They found that the parameters that reproduce the QM potentials well can still lead to wrong ligand conformations in MD simulations. Furthermore, they showed that there can be multiple sets of parameters that reproduce the quantum potentials equally well, but only one set of parameters can reproduce the desired geometry during MD simulations. Similarly, Pang et al. recently reported their optimisation protocol for the drug molecule AT130 [22] with a halogen $\sigma$-hole particle, where they proposed a method for finding a proper set of dihedral multiplicities via fitting the dihedral angles one by one. These examples illustrate the relevance of finding the correct dihedral multiplicity (*n*) as well as introducing extra multiplicities to improve the fitting results. In contrast, the other crucial parameter of the dihedral parametrisation, the dihedral phase shift $\delta$, has relatively rarely been addressed. So far, the only well established rule is that for the double bond phase shift one should set $\delta =180^\circ$ for $n=2$ [22].

Optimising ring structures is often considered very challenging, especially when multiple dihedral rotations are coupled. One example is the norfloxacin parametrisation via ParaMol [23], reported by Morado et al. recently [18]. In this study, an extensive 2-dimensional (2D) potential energy surface (PES) scan had to be performed, as the corresponding 1D scans resulted in severe discontinuities of the energy profile, reflecting a flip between two distinct low-energy structures. The same situation is also encountered for $\beta$-lactams: As we shall see in the Results section, when performing the parametrisation for penam, we observe a ring flipping that is energetically accessible by a simultaneous rotation of two single bonds. In our case however, the difficulty doesn’t come from fitting to only the 1D dihedral potentials, but rather, blindly optimising the parameters leads to a uselessly good dihedral potential fit that yields a wrong conformation during the associated MD simulation. For instance, in the case of penam analogue, naively fitted dihedral parameters lead to a wrong MD conformation which deviates strongly from the optimised quantum geometry (RMSD of 0.65 Å); see the Results section for details. To achieve a proper parametrisation of $\beta$-lactams and consequently enable future MD studies, we start with the simplest $\beta$-lactam structure, monobactam (also known as 2-azetidinone), and discuss the impact of the dihedral phase shifts on the $\beta$-lactam parametrisation.

## Method

**Fig. 2:**
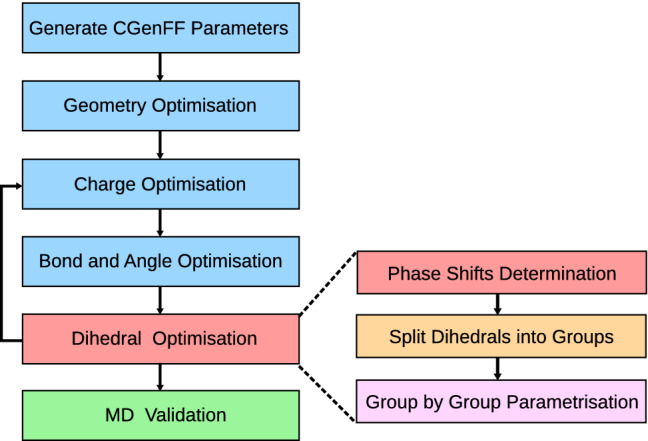
The parametrisation workflow. **Generate CGenFF parameters**: The initial CGenFF parameters are predicted using the ParamChem webserver. **Geometry Optimisation**: The equilibrium geometry is determined via quantum calculations. **Charge Optimisation**: The atom charges are fitted by reproducing the quantum interaction energy with water, the quantum equilibrium distance, and the quantum dipole moment. **Bond and Angle Optimisation**: Force constants and equilibrium bond lengths/angles are determined by fitting distortion energies calculated from the quantum Hessian matrix. **Dihedral Optimisation**: We introduce a phase shift determination step into the traditional ffTK workflow (see text for details). The phase shifts are fixed during the potential fitting, unless mentioned otherwise. As the phase shifts sometimes can be ambiguous, different sets of parameters should be recorded and validated by MD simulations. Finally, a second iteration of the parameter optimisation is carried out to ensure convergence

The ffTK workflow [17] for optimising parameters of a ligand is depicted in Fig. 2. The initial guess of the parameters is generated by the CGenFF ParamChem webserver [15, 16, 24]. Based on the CGenFF results, one then decides which parameters to optimise. Normally only parameters with penalties larger than 10 need to be optimised, and each optimisation step contains 2 tasks: Performing quantum mechanics (QM) calculations as well as fitting the molecular mechanics (MM) parameters to reproduce the QM results. Most QM calculations are performed at the MP2/6-31G(d) level, with extra diffuse functions for anionic molecules, i.e. the 6-31+G(d) basis set. The only exception occurs when one calculates the water interaction for the charge optimisation. Because CGenFF determines the atom charges based on the interaction energy calculated at the HF/6-31G(d) level, using a different basis set for the anion can lead to unusually large charges that deviate from the usual CGenFF charge range. Out of curiosity, we tried HF/6-31+G(d) for the oxacephem anion interacting with water. This leads to fitted atom charges very close to $+1$ or $-1$. The problem remains when using MP2/6-31+G(d). Therefore, throughout this work we follow the original protocol and use HF/6-31G(d) in the charge optimisation for anions.

There are a few things to note in the workflow. First, the purpose of the geometry optimisation is to find the minimum energy structure along all dihedral rotations, i.e. finding the global minimum of the structure. Therefore, we normally perform the quantum torsion scans immediately after the initial quantum geometry optimisation. If any lower energy conformation is found during the torsion scan, we update the initial structure and repeat the geometry optimisation and torsion scans until the global minimum is reached. Eventually, the quantum optimised geometry will serve as the target equilibrium geometry for the MD validation. Second, we update all force field parameters except for the partial atomic charges, where only non-hydrogen atoms with penalties larger than 10 are optimised. We are motivated to do so because CGenFF provides some angle parameters with zero penalties, but their MM equilibrium angles are far from the optimised quantum geometry. (For details see Results section, monobactam.) Therefore, we optimise *all* bonded parameters, including bonds, angles, and dihedrals, regardless of their reported penalties. All our optimised bond lengths and angles are found to lie within 0.03 Å and 3$^\circ$ of the optimised quantum geometry, respectively. Third, the dihedral scans are performed for all dihedral angles, including those involving hydrogen atoms. All these data are used for the parametrisation, but only data for selected dihedrals (i.e. those without hydrogen atoms) will be presented. In general, the relaxed potential energy scans (with geometry optimisation at each point) are performed for $\pm 90^\circ$ from the equilibrium geometry, with a step size of $10^\circ$. Dihedrals with all atoms on the 4-member ring are scanned for $\pm 45^\circ$ with a step size of $5^\circ$. Some dihedrals are scanned up to $\pm 120^\circ$ or $\pm 150^\circ$ in order to provide data points below the energy cutoff of 10 kcal/mol. The same holds for the carboxylate rotation, where $\pm 180^\circ$ degrees are scanned.

The dihedral phase shifts can be determined from the optimised quantum geometry: Since we begin with a global minimum and use an additive force field, the dihedral phase shifts should be chosen such that the local minimum of the cosine terms in Eq. 1 coincides with the optimised geometry. In practice, this is done by directly comparing the dihedral potentials calculated using both $\delta =0^{\circ }$ and $\delta =180^{\circ }$. Whichever returns a lower potential value will be taken as the associated phase shift. Hereafter, we will refer to this choice of $\delta$ as the “default” choice. However, occasionally the two values will be very close, so that the choice for the dihedral phase shift becomes ambiguous. Thus, validating parameter sets with different $\delta$ via MD simulations is necessary.

Figure 2 also shows our protocol for determining the dihedral parameters. As the $\beta$-lactam cores are composed of fused rings, we further split the dihedral angles into a few groups, e.g. one group for the 4-member ring, one group for the 5/6-member ring, etc., and optimise parameters within each group. The 4-member ring group contains the dihedrals that involve rotations of the 4 single bonds on the ring, while the rest are grouped as the fused 5/6-member ring dihedrals. The carboxylate and the improper dihedrals are usually updated at the end.

Finally, the quantum calculations in this work are performed with Gaussian [25]. The MD simulations in this work are performed using Gromacs [26–28]. The ligand is solvated in a cubic water box, with a distance of 1.5 nm from the ligand to the box. For anionic ligands, one sodium ion is added to neutralize the system. Following the CHARMM force field standard, the van der Waals force switching is turned on at 10 Å and cuts off completely beyond 12 Å. The electrostatic interaction is calculated using the Particle Mesh Ewald (PME) method [29], with a spline order of 4 and a grid spacing of 1.25 Å. The temperature and pressure are controlled at 300 K and 1 bar using the V-rescale thermostat [30] and Parrinello-Rahman pressure coupling [31, 32], respectively. Bonds involving hydrogens are constrained using LINCS [33]. Prior to the 50 ns MD simulation, the system is first subjected to a 50,000 step energy minimization to remove any high energy clashes, a 100 ps NVT equilibration to heat up the system, and a 100 ps NPT equilibration to equilibrate the pressure. Conformations of the ring structure without hydrogen atoms nor the carboxylate group are clustered, using the GROMOS method [34] with a 0.2 Å RMSD cutoff. The cluster centroid, which is a frame from the MD trajectory, is taken as the representative for the cluster. The associated conformation is then compared with the optimised quantum geometry and the corresponding RMSD is reported.

## Results

### Naive parametrisation without dihedral phase shift prediction

**Fig. 3:**
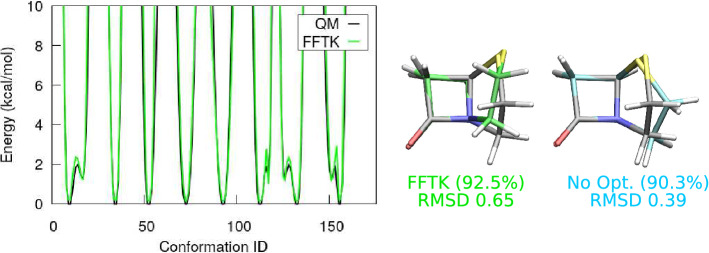
Results of a naive parametrisation for penam analogue. Depicted are selected potential curves obtained from quantum torsion scans (QM) and from the MM parameters (FFTK), where the dihedral phase shifts were determined by ffTK during the potential curve fitting. Conformations of the major cluster from MD simulations using this FFTK parameter set (green) and using the non-optimised CGenFF parameters (cyan) are compared with the optimised quantum geometry (gray). The size of the cluster is reported as a percentage of the length of the MD trajectory, and the RMSD is given in Å

We begin our discussion with a naive parametrisation of the penam analogue, i.e. the penam molecule without the carboxylate group, see Fig. 1 for the full structure. Following the traditional approach, we let ffTK determine all parameters for the dihedral angles, including their phase shifts $\delta$. That is, the $\delta$ values are chosen such that the difference between the MM potential and the QM potential is minimized. In Fig. 3, the MM potentials obtained in this way are labelled as “FFTK”, and they agree well with the quantum dihedral potentials. However, the MD simulation performed using these “FFTK” parameters shows a major molecular conformation which differs dramatically from the optimised quantum geometry, with a huge RMSD of 0.65 Å. In particular, the 4-member ring from the MD conformation cannot be aligned properly with the corresponding quantum structure. For comparison, we also performed the MD simulation using the parameters generated by the CGenFF webserver directly, without further optimisation. As expected, 90.3$\%$ of the associated trajectory show a conformation different from the quantum geometry, but with a better RMSD of 0.39 Å. While the non-optimised parameters with high penalties are expected to be problematic, they at least lead to a major conformation where the 4-member $\beta$-lactam ring compares well with the quantum geometry. On the other hand, the naive parametrisation yields a conformation where both the 4-member ring and the 5-member ring are distorted, suggesting that a naive parametrisation may be worse than not doing any optimisation at all. This observation prompts us to reconsider the parametrisation process for the dihedral angles more carefully.

### Monobactam

**Fig. 4:**
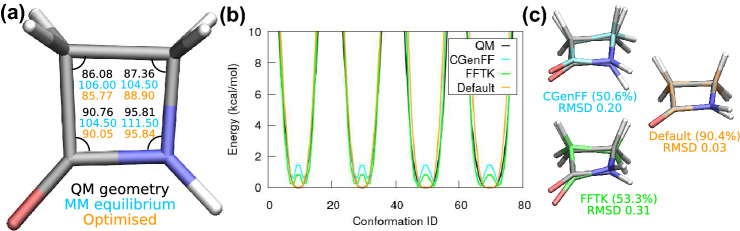
(a) Optimised quantum geometry of monobactam. The equilibrium angles from the CGenFF-supplied MM parameters (cyan) deviate greatly from the quantum geometry (black). Our optimised angle values are listed in orange. (b) Dihedral potential fitting using different choices of phase shifts. Shown are dihedrals without hydrogen atoms. Quantum potentials are depicted in black. Potentials fit using the CGenFF phase shifts (cyan) or letting ffTK find the $\delta$s (green) both show a strange energy barrier at the QM potential minimum. Only the $\delta$s determined from the optimised quantum geometry (orange) can fit the QM potential well. (c) The major conformation obtained from 50 ns MD simulations using parameters shown in panel (b). The cluster size (as a percentage of the trajectory length) and the RMSD (in Å) compared with the quantum geometry are also listed. As their potential fits exhibit strange barriers, the FFTK and CGenFF MD conformations do not agree with the quantum geometry. In contrast, the default phase shifts (labeled as “Default” ) reproduce the quantum geometry well, with only hydrogen atoms showing a small deviation

Because it is difficult to tell which part of the workflow leads to the bad parametrisation of the fused rings, we start by looking for clues with monobactam. Monobactam is the simplest $\beta$-lactam, and its parameters were validated and included in the original CGenFF database. Therefore, the CGenFF webserver shows zero penalties for these parameters. These parameters are also used as the initial guess for other $\beta$-lactams, and their penalties are still reported as zero. While the optimised quantum geometry of monobactam can be reproduced well using the original CGenFF parameters, the associated QM and MM equilibrium angles of the 4-member ring differ significantly by up to $20^\circ$, see Fig. 4a. Although the MM values resemble the proper equilibrium angle of an sp$^3$ hybrid orbital for a single bond ($109^\circ$), these values raise concern about the underlying approximation employed. Namely, the bonds and angles are modeled as harmonic oscillators, vibrating around the optimised quantum geometry. Under this assumption, an equilibrium bond length or angle value is expected to be within 0.03 Å or 3$^\circ$ of the optimised quantum geometry [14]. Hence, we decide to optimise the parameters of all bonds and angles, including those without penalties. The optimised values agree well with the optimised quantum geometry, as shown by the orange numbers in panel (a).

A major challenge occurs when we try to fit the dihedral potential energy curves to the data from the quantum torsion scans. Again, we let ffTK decide which $\delta$ value to take during the potential fitting, and we label this choice as “FFTK”. Additionally, we tried the approach of keeping the phase shifts the same as those given by the webserver (labeled as “CGenFF”). However, as shown in Fig. 4b, neither the FFTK nor the CGenFF choice can reproduce the QM potentials. Surprisingly, for both of these choices, the resulting MM potentials exhibit an energy barrier right in the middle of the QM potential minima. The associated MD conformations hence differ significantly from the optimised quantum geometry (RMSD > 0.2 Å). In contrast to the FFTK and the CGenFF phase shifts, if we predict $\delta$ based on the optimised quantum geometry (labeled as “Default”), then the quantum potentials are reproduced well, and the associated MD simulation yields a major conformation nearly identical with the quantum one (RMSD $\sim$ 0.03 Å). This indicates that predicting the phase shifts prior to the potential fitting is the right approach. See Fig. 4c for the MD conformations obtained using the FFTK, the CGenFF, and the Default phase prediction.

### Penem, oxapenem, carbapenem

**Fig. 5:**
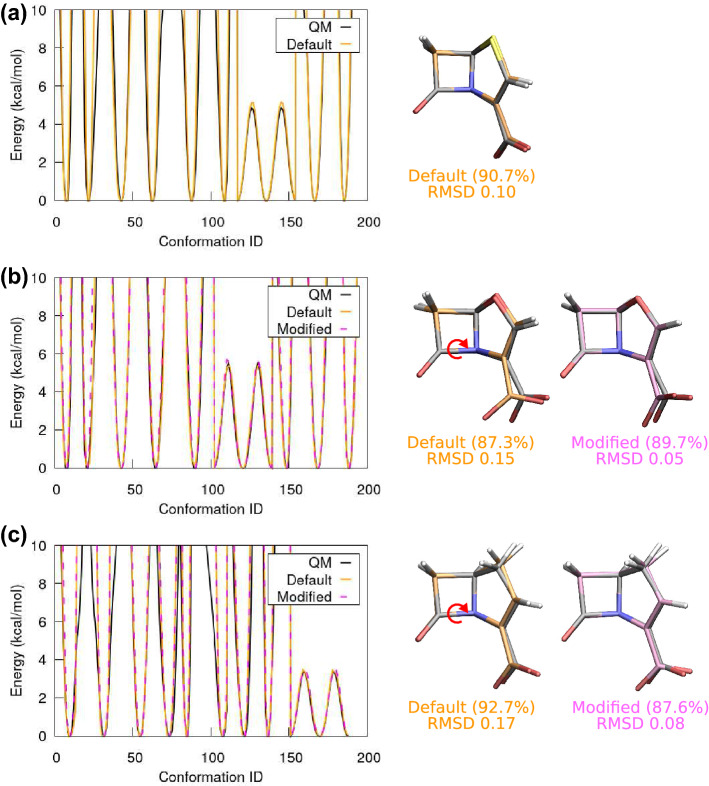
(a) Selected results of dihedral potential fitting and the associated MD major cluster for penem. Phase shifts $\delta$ are determined based on the optimised quantum geometry. Good agreement with quantum data is found. (b) Selected results of potential fitting for oxapenem. Shown are results with $\delta$ predicted from the quantum geometry, and with modified parameters where two $\delta$s have been changed from $0^{\circ }$ to $180^{\circ }$. The modified dihedrals are associated with the C-N single bond rotation, indicated by the red arrow. This modification results in better agreement with the quantum geometry. The RMSD is as low as 0.05 Å. (c) Selected results of potential fitting for carbapenem. Similar to oxapenem, modifying the two $\delta$s yields a better result than the default $\delta$. The RMSD is only 0.08 Å

After successfully parametrising monobactam, we turn to the group of penems, using the same protocol. Again, we optimise the atomic charges as well as the MM parameters for all bonds and all angles. Since these operations are well explained in the literature [14, 17, 22], the only thing to note is that one should employ the 6-31G(d) basis set when calculating the water interactions for the anionic ligands. Following the same protocol as in monobactam, we complete the dihedral potential fitting to obtain the MM parameters (labeled as Default), and perform the MD simulations to validate the conformations. Results for penem, oxapenem, and carbapenem are depicted in Fig. 5, panel (a), (b), and (c), respectively. For penem, the MM potentials calculated using the default phase shifts reproduce the quantum dihedral scans, and the conformation of the major cluster, obtained from the 50 ns MD simulation, matches the quantum geometry. However, for oxapenem and carbapenem the same phase shift determination method results in major clusters that don’t match their quantum geometries very well, even though the corresponding potentials are good fits to the quantum curves, cf. the black and orange curves in Fig. 5(b) and Fig. 5(c).

Why does our $\delta$-prediction perform worse for the last two ligands? While the prediction based on the optimised quantum geometry is certainly a good initial guess for $\delta$, sometimes this prediction may be ambiguous owing to the size of atoms. Recall that the only difference between penem, oxapenem, and carbapenem is that one site is either a sulfur atom, an oxygen atom, or a methylene (-CH$_2$-) group, respectively. These moieties have the same number of valence electrons, therefore structures of the three ligands should be very similar, and so are their MM dihedral parameters (*n* and $\delta$). However, the difference in atomic size leads to an offset in their optimised quantum geometries, and consequently a few phase shifts determined from that geometry differ. Indeed, two dihedral angles associated with the C-N single bond rotation (indicated in Fig. 5) have $\delta =180^\circ$ in penem but $\delta =0^\circ$ in oxapenem and carbapenem. Since the corresponding phase shift in monobactam is also $180^\circ$, it is likely that the predicted default phase shifts for these two dihedrals in oxapenem and carbapenem are suboptimal. After these two $\delta$s are modified, the MD simulation immediately recovers the optimised quantum geometry, see the magenta structures in panel (b) and (c), and the quality of the potential fits remains the same, cf. the magenta dashed and orange curves. We conclude that the phase shifts associated with this C-N single bond should be set to 180$^\circ$ for all $\beta$-lactams, and this will be used as the default $\delta$ in all below examples.

### Penam, oxapenam, carbapenam

**Fig. 6:**
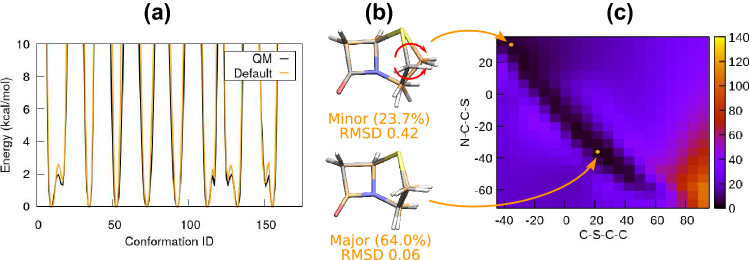
Results for penam analogue. (a) Selected potentials obtained from 1D quantum torsion scans. The QM potentials are reproduced by fitted MM potentials, obtained with the default phase shifts (Default). (b) The major and minor cluster conformations obtained from MD simulation, using the fitted MM potentials. The cluster size as well as the RMSD in Å compared to the quantum geometry (black) are given. The two conformations can interchange through a simultaneous rotation of two single bonds, indicated by the red arrows in the minor conformation. (c) The potential energy surface obtained from a 2D quantum torsion scan. The major and minor conformations are located at $(21.7^\circ , -36.2^\circ )$ and $(-35.6^\circ , 31.1^\circ )$, respectively, and shown by the orange dots in the plot

Next we consider the group of penams. This group includes penam, oxapenam, carbapenam, and the penam analogue discussed previously. For simplicity, we begin with the penam analogue. In contrast to what was shown in the beginning of the Results section, if we use the default $\delta$ determined from the optimised quantum geometry, the QM potentials and geometry of penam analogue are reproduced well, see Fig. 6(a) and (b). Interestingly, the major MD conformation now only represents 64.0 $\%$ of the trajectory, and a minor conformation representing 23.7 $\%$ of the trajectory warrants attention. Compared to the major one, the minor conformation has its 5-member ring flipped over. This can be achieved by simultaneously rotating two single bonds on the 5-member ring, as indicated by the red arrows in Fig. 6(b). A scan of the 2-dimensional (2D) potential energy surface further supports the feasibility of such a motion: as shown in Fig. 6 (c), there is a potential valley diagonally across the 2D quantum potential energy surface, connecting the major and minor conformations. Clearly, if the two dihedrals rotate simultaneously, there is almost no energy barrier for the conformational change.

**Fig. 7:**
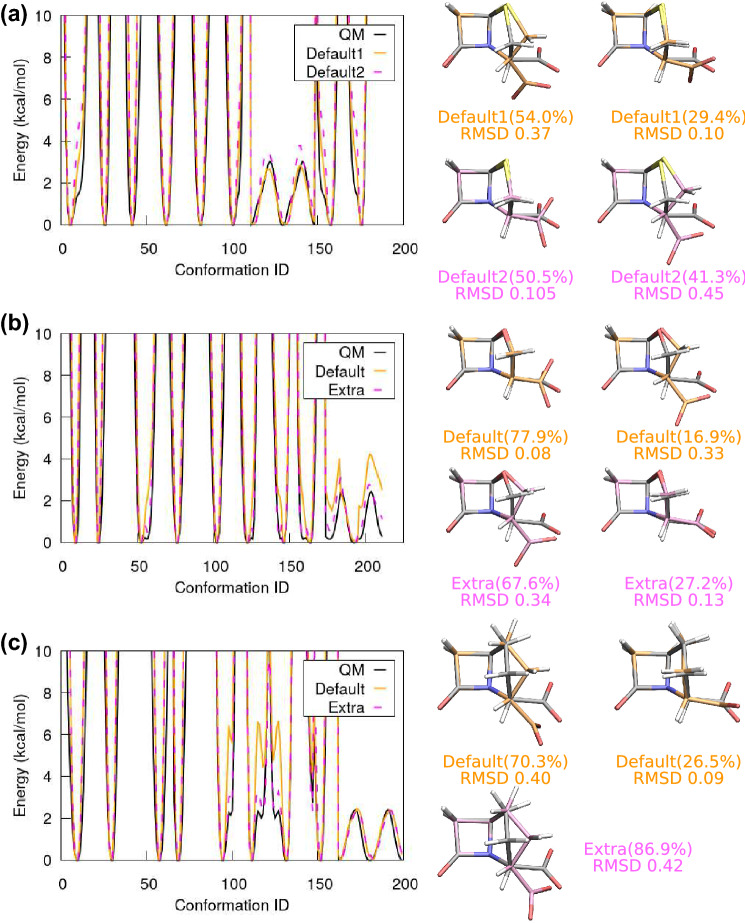
(a) Selected dihedral potentials of penam, obtained by quantum calculations (black) and by MM parameter fitting, i.e. Default1 (orange) and Default2 (magenta). The two Default sets have identical *n* and $\delta$, but different $k_{\phi }$. MD simulations using the two MM parameter sets show distinct outcomes: Default2 has the quantum geometry as the major conformation (magenta), while Default1 has it as the minor conformation (orange). (b) The dihedral fitting of oxapenam and the associated MD conformations. While the Default potential fit is not too good, it leads to a set of parameters that reproduces the optimised quantum geometry during the MD simulation. Introducing extra dihedral multiplicities (extra *n*) improves the potential fit, but the quantum geometry now only represents 27.2$\%$ of the MD trajectory. (c) The dihedral fitting of carbapenam and the associated MD conformations. The default $\delta$ without extra multiplicities does not reproduce the quantum potentials for the 5-member ring flipping very well, but the associated MD simulation finds two conformations: a flipped structure (70.3$\%$) and the optimised quantum geometry (26.5$\%$). Upon introducing extra multiplicities, the dihedral fitting quality is improved, but the quantum geometry is no longer found in the MD trajectory

While parameterising the penam analogue now becomes rather straightforward, the actual penam with the carboxylate group requires a few additional considerations. First, parameters for penam are already available in literature [35], but the reported multiplicities are very different compared to CGenFF. Here we follow the CGenFF multiplicities for consistency, unless they seemed unreasonable. For example, the carboxylate group has a two-fold rotational symmetry, so we change the associated multiplicities to $n=2$ instead of using the $n=3$ suggested by CGenFF. The most significant issue is encountered when parameterising the 5-member ring: Namely, we obtained two sets of dihedral parameters with similar quality, which share the same values for the multiplicities *n* and phase shifts $\delta$, but differ only in the force constants $k_{\phi }$. Shown in Fig. 7(a) are the dihedral results for these two parameter sets, labeled as “Default1” and “Default2.” Both of them reproduce the quantum potentials with similarly good quality. However, the associated MD simulations show opposing trends: The optimised quantum geometry corresponds to the minor cluster ($\sim 29.4\%$) in Default1 but to the major cluster ($\sim 50.5\%$) in Default2. Judging only by these results, one may conclude that Default2 is a better choice of parameters, as it reproduces the quantum structure as the major cluster. On the other hand, 2D dihedral potential scans (not shown here) look similar to the penam analogue 2D scan, which indicates that the 5-member ring flipping is a universal behaviour across penam, oxapenam, and carbapenam. It makes sense to postpone a conclusion about which parameter set is better until we see the fitting results for the other two, i.e. to find a consistent trend across all three ligands.

**Fig. 8:**
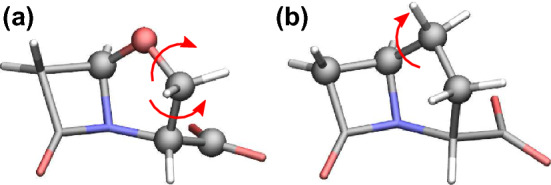
(a) Dihedral angles of oxapenam that require extra multiplicities. For both angles, the term with $n=4$, $\delta =0$ is added. Notably, $\delta =0$ matches the predicted phase shifts using the flipped structure. (b) The dihedral rotation in carbapenam with two extra multiplicities: $n=1$, $\delta =0$ and $n=4$, $\delta =0$

Results for oxapenam are shown in Fig. 7(b). The MM parameters based on our default $\delta$ prediction (labeled as “Default”) reproduce the quantum geometry as the major (77.9$\%$) MD conformation, but the quantum potentials associated with the 5-member ring flipping are not reproduced well, cf. the orange and black curves. This directly affects the quality of dihedral fitting for the carboxylate group, because its rotation is also coupled with the 5-member ring flipping. To improve the potential fits, we introduce extra multiplicities for the two dihedral angles depicted in Fig. 8(a), namely a potential term with $n=4$ and $\delta =0$ is added for both angles. These extra dihedral multiplicities are introduced according to Y.T. Pang’s protocol [22], namely extra *n* are introduced one by one, and we then collect those that substantially improve the potential fits. Interestingly, the phase shifts for these extra *n* correspond to the flipped ring structure, i.e. to the other energy minimum that is nearly degenerate with the optimised quantum geometry. Indeed, we find that introducing the additional dihedral multiplicities improves the quality of the potential fits. Furthermore, the quantum geometry now becomes the minor MD conformation ($\sim 27.2\%$), which is in line with the previous result for penam with “Default1” parameters. See results labeled “Extra” in Fig. 7(b). A similar situation is found for carbapenam as well. Fig. 7(c) shows the MM and QM dihedral potential curves, as well as the major and minor MD conformations. The default MM parameters do not reproduce the quantum potential perfectly, but the quantum geometry is captured by the minor MD cluster with a similar percentage as in penam and oxapenam (26.5$\%$). Upon introducing extra multiplicities for the angle shown in Fig. 8(b), the quality of the potential fit is improved, but the MD simulation is now dominated by the flipped 5-member ring structure ($86.9\%$) and no longer finds the quantum geometry. Since the 2D dihedral scan indicates that the 5-member ring flipping should occur naturally, parameters with the extra multiplicities are considered a bad choice for carbapenam.

Across the three molecules in the penam group, a consistent trend is seen: The 5-member ring flipping is a common behaviour in the penam group. When there is no carboxylate group, i.e. for penam analogue, the MD simulation spends about a quarter of the time in the flipped structure. Once the carboxylate group is introduced, i.e. for penam, oxapenam and carbapenam, the MD simulation spends only about a quarter of the time in the unflipped, optimised quantum geometry. Therefore, we conclude that such a ring flipping should be allowed, and the parameter sets that preserve this trend are the better choice. We stress that this 5-member ring flipping is a dynamic process. It occurs frequently and allows the ligand structure to change back and forth between the major and minor conformations. Such a motion is also observed in molecules from other groups, but with a lower frequency, as the double bond on the 5-member or 6-member ring increases the difficulty of ring flipping.

### Cephem, oxacephem, carbacephem

**Fig. 9:**
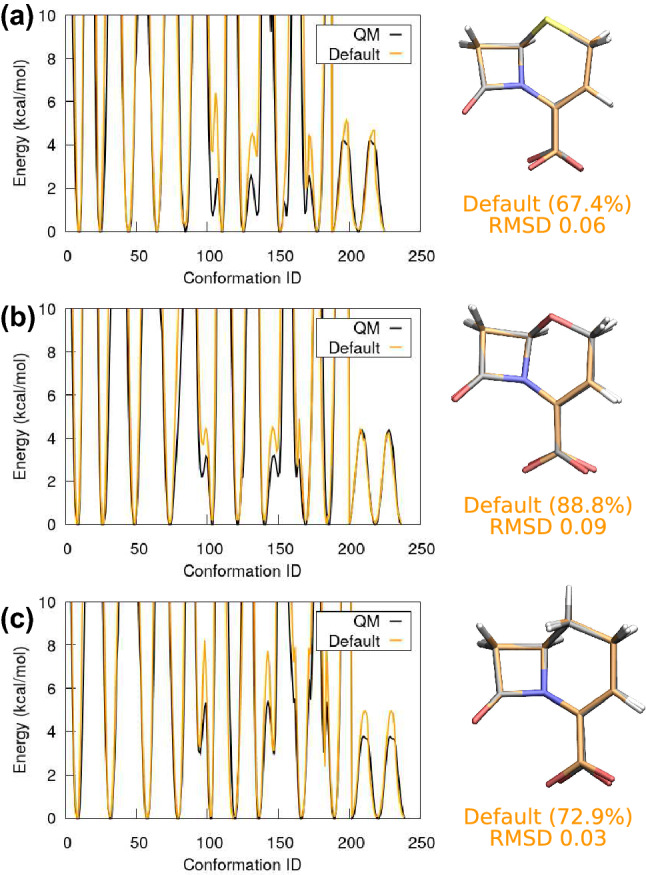
(a) Selected dihedral potentials of cephem and the associated major MD conformation. The MM potential grasps the 6-member ring dihedral rotation qualitatively. Nevertheless, the major MD conformation reproduces the optimised quantum geometry well (shown in black). (b) Selected dihedral potentials of oxacephem and its major MD conformation. In comparison with cephem, the MM potential fits the QM result better, and the MD major conformation also represents a larger portion of the simulation, viz. 88.8$\%$ instead of 67.4$\%$. (c) Selected dihederal potentials of carbacephem and the major MD conformation. The major features of the quantum results are reproduced

The last group comprises cephem, oxacephem, and carbacephem. The skeleton of these molecules is a 4-member ring fused with a 6-member ring. Additionally, there is a double bond on the 6-member ring. As one would expect, dihedral force constants $k_{\phi }$ associated with the double bond rotation are very high. In contrast, the other 6-member ring dihedrals generally have lower force constants. When employing ffTK for dihedral parametrisation, it is therefore natural to further separate the dihedrals into different groups: One associated with the double bond rotation and one with the single bond rotations. While such a strategy may not always be necessary, when optimising all the 6-member dihedral parameters simultaneously for cephem and oxacephem, we observed that the largest force constant occurs on a single bond instead of the double bond. This, of course, contradicts the common sense about how difficult it is to rotate the bonds. Hence, for these two molecules, we first fit the double bond dihedrals and then fit the rest of the 6-member ring dihedrals. The carboxylate rotation and improper rotation are then parameterised at the end, based on the updated 6-member ring parameters. Default phase shifts based on the optimised quantum geometry are employed for all three molecules. Good results are obtained, as shown in Fig. 9(a), (b), and (c) for cephem, oxacephem, and carbacephem, respectively. Our protocol yields parameters that result in a good major MD conformation reproducing the optimised quantum geometry, although the MM potentials of the 6-member ring are a bit off from the quantum data. Nevertheless, the relevant features of the quantum potentials are all captured, cf. the black and orange curves in Fig. 9.

## Conclusion

In this work we optimised the CGenFF MM parameters for monobactam, penam analogue, and 9 other $\beta$-lactams, using the force field Toolkit (ffTK) and Gaussian calculations. While CGenFF provides a good initial guess for the dihedral multiplicities, determining the dihedral phase shifts by the potential curve fitting often leads to bad parameters that cannot reproduce the optimised quantum geometry during MD simulations. To resolve this problem, here we introduced a step of dihedral phase shift prediction into the ffTK optimisation protocol, and successfully parameterised most of the molecules, including monobactam, penem, penam analogue, penam, carbapenam, cephem, oxacephem, and carbacephem. With minor modifications, such as changing selected phase shifts or introducing additional multiplicities, we were also able to successfully parametrise oxapenem, carbapenem, and oxapenam. Overall, the MM results obtained with our optimised parameters are in excellent agreement with the quantum data: The dihedral potential curves are reproduced, and the optimised quantum geometry is strongly represented in associated molecular dynamics simulations. Our results demonstrate that predicting the dihedral phase shifts can be the key to a successful parametrisation, and therefore this step should be incorporated into the workflow for optimising dihedral parameters.

## Supplementary Information

Below is the link to the electronic supplementary material.

Supplementary file1 (.zip 40.0 KB)

## Data Availability

The optimised charges and parameters are available as str files in the supplementary information to this article online. Other data are available from the corresponding author upon reasonable request.
